# Post-Operative Recurrent Trachomatous Trichiasis Is Associated with Increased Conjunctival Expression of *S100A7* (Psoriasin)

**DOI:** 10.1371/journal.pntd.0001985

**Published:** 2012-12-20

**Authors:** Matthew J. Burton, Saul N. Rajak, Athumani Ramadhani, Helen A. Weiss, Esmael Habtamu, Baye Abera, Paul M. Emerson, Peng T. Khaw, David C. W. Mabey, Martin J. Holland, Robin L. Bailey

**Affiliations:** 1 London School of Hygiene and Tropical Medicine, London, United Kingdom; 2 Kilimanjaro Christian Medical Centre, Moshi, Tanzania; 3 NIHR Biomedical Research Centre, Moorfields Eye Hospital and UCL Institute of Ophthalmology and UCL Partners AHSC, London, United Kingdom; 4 The Carter Center, Bahir Dar, Ethiopia; 5 Bahir Dar University, Bahir Dar, Ethiopia; 6 The Carter Center, Atlanta, Georgia, United States of America; University of California San Diego School of Medicine, United States of America

## Abstract

**Background:**

Surgery for trachomatous trichiasis (TT) is a key component of the SAFE Strategy for trachoma control. Unfortunately, recurrent TT following surgery is common, probably due to various surgical and disease factors. To develop strategies to reduce recurrence rates it is necessary to understand its pathological basis. In this study we investigated the relationship between recurrent trichiasis and the expression of various cytokines and fibrogenic genes during a two-year follow-up period.

**Methodology/Principal Findings:**

Individuals undergoing surgery for TT were examined at baseline (pre-operative), 6, 12, 18 and 24 months. Conjunctival swab samples were collected from the tarsal conjunctiva for RNA isolation on each occasion. Individuals who developed recurrent TT with at least 3 lashes touching the eye on one or more occasion were designated “cases” and an equal number of “controls” were randomly selected from those without recurrent TT, frequency matched for age and baseline TT severity. The expression of the following genes was measured by quantitative RT-PCR: *S100A7, IL1B, CXCL5, TNFA, NOS2A, CTGF, MMP7, MMP9* and *MMP12*. Thirteen hundred individuals were enrolled and underwent surgery. By two years 122 had developed recurrent TT with at least 3 lashes touching the eye. Recurrent TT was consistently associated across multiple time points with about a 2-fold increase in *S100A7* expression (p = 0.008). Clinically visible conjunctival inflammation was associated with increased *S100A7, IL1B, CXCL5, MMP9* and *MMP12* expression.

**Conclusions/Significance:**

Increased *S100A7* expression was associated with trachomatous conjunctival scarring and may be linked to the pathophysiology of recurrent TT. *S100A7* expression could be a potential biomarker for this disease process. As part of the epithelial innate immune response S100A7 has multiple actions, potentially contributing to a chronic pro-inflammatory response, which may lead to ongoing tissue damage and increased scarring.

## Author Summary

Trachoma causes blindness through corneal damage from in-turned eyelashes (trachomatous trichiasis [TT]). Trichiasis is treated surgically to correct the anatomical abnormality. Unfortunately, TT frequently returns following surgery, which again puts the person at risk of sight loss. Recurrent trichiasis is multifactorial. We investigated the possible role of various immuno-fibrogenic factors. To do this we operated on 1300 people with TT and followed them up every six months for a two-year period. On each occasion a conjunctival swab was collected for human gene expression analysis. We measured various factors that are thought to be important in inflammation and scarring diseases. The gene expression profile of people who developed recurrent TT was compared to a sample of those that did not have a recurrence. Recurrent TT was associated with increased expression of psoriasin (*S100A7*) before surgery and on multiple occasions during a two-year follow-up period. S100A7 is able to promote inflammation and may contribute to the development of the scarring process in trachoma.

## Introduction

Trachoma remains the leading infectious cause of blindness worldwide [1]. Trachomatous Trichiasis (TT) is the potentially blinding stage of this disease in which the eyelashes scratch the surface of the globe, resulting in corneal opacification [2]–[4]. It is the result of a progressive scarring process of the tarsal conjunctiva, which is initiated in childhood by recurrent episodes of *Chlamydia trachomatis* infection that are characterised by inflammation. However, the pathophysiology of this scarring process is poorly understood; it is unclear which immuno-fibrogenic mechanisms are most important and what factors drive disease progression, particularly in the late stages when *C.trachomatis* can rarely be detected [2], [5].

Surgery is performed to correct TT. Unfortunately, TT frequently recurs, particularly when performed under operational conditions [2], [6]–[8]. Recurrent TT is a significant problem in preventing blindness from trachoma, undermining the potential success of surgical programmes. Prospective long-term data suggest that TT recurrence can be broadly subdivided into two phases: early and late [2], [9]–[12]. Early recurrence, which may be defined as that developing within the first six months following surgery, appears to occur with a greater incidence rate than late recurrence [9].

Early recurrence is probably attributable to a combination of factors including pre-operative disease severity, how well the surgery was performed, and possibly early post-operative wound healing responses [2], [13]. Early trichiasis recurrence rates may be reduced by improving the quality of surgery and refining the operative procedure [8], [14]. Inter-surgeon variation in recurrence rates has been reported, suggesting that operative factors can significantly affect results [2], [12], [15]. Currently, the World Health Organization (WHO) endorses the bilamellar and posterior lamellar tarsal rotation procedures (BLTR and PLTR) [8], [16]. The BLTR has been the subject of several clinical trials comparing it to alternative procedures (other than the PLTR) and was found to give the best results [10], [17], [18]. Currently, it is not know whether the BLTR and PLTR are equally effective. There may also be scope for improving results through influencing the course of the post-operative wound healing process by regulating excessive contractile scarring, analogous to glaucoma filtration surgery. However, these early biological events have yet to be studied.

Late recurrence is likely due to immune-mediated conjunctival scarring disease, however, the pathological basis of progressive cicatrisation in trachoma remains to be elucidated. It may be driven by recurrent infection, yet, it is relatively unusual to detect *C. trachomatis* infection in people with scarring trachoma, and it has not been found to be a risk factor for recurrent TT [2], [5], [6], [11], [19]. In contrast, other non-chlamydial bacterial pathogens are identified relatively frequently and have been associated with recurrent trichiasis [2], [19], [20]. We have previously found such infections to be associated with altered gene expression patterns for inflammatory cytokines and tissue modifiers such as matrix metalloproteinases (MMP) [20], [21]. The potential of post-operative oral azithromycin to reduce recurrence has been investigated. In one trial from Ethiopia, a region with generally high *C. trachomatis* prevalence, recurrence was reduced, although this was not linked to *C. trachomatis* [11]. In a separate trial from The Gambia with a lower prevalence of *C. trachomatis*, post-operative azithromycin did not affect the recurrence rate [2]. Similarly, in a study from Nepal, the recurrence rates were comparable in the azithromycin and placebo groups [22].

A better understanding of the immuno-fibrogenic basis of recurrent TT may help in developing interventions that reduce recurrence that is attributable to the scarring disease process. We have previously examined the conjunctival transcriptome in scarring trachoma in a case-control study [5]. This identified various factors that are differentially expressed in scarred conjunctival tissue compared to normal controls: pro-inflammatory cytokines and several MMPs were particularly prominent. The gene with the largest relative increase in expression was the pro-inflammatory, anti-bacterial peptide *S100A7* or Psoriasin, which was increased 15-fold in trichiasis cases before surgery, compared to controls [5]. We have also previously reported the conjunctival expression level of several factors at a single time point one year after TT surgery and found recurrent TT was associated with a reduced *MMP1*/*TIMP1* ratio [20].

Recently we reported the results of a randomised controlled trial of absorbable (vicryl) versus silk sutures for TT surgery; there was no difference in outcome between the two treatment arms [12]. During this trial we re-examined participants at six-monthly intervals for two years. On each occasion conjunctival swab samples were collected for gene expression analysis. Here we report the expression of various pro-inflammatory cytokines and MMPs in a longitudinal comparison of trial participants with and without recurrent TT. We examined the hypothesis that some of these factors are linked to recurrent disease and sought to identify biomarkers associated with recurrent TT.

## Methods

### Ethics statement

This study adhered to the tenets of the Declaration of Helsinki and was approved by three science and ethics committees: the National Health Research Ethics Review Committee, Ministry of Science and Technology, Ethiopia, the London School of Hygiene and Tropical Medicine Ethics Committee (UK) and Emory University Institutional Review Board (Atlanta, USA). Potential participants were provided with both written and oral information in Amharic about the trial. For those agreeing to participate, written informed consent in Amharic was required prior to enrolment. If the participant was unable to read and write, the information sheet and consent form were read to them and their consent recorded by witnessed thumbprint, which was approved by the Ethics Committees.

### Participant selection and surgical treatment

This nested case-control study was conducted within a surgical trial of absorbable versus silk sutures in the treatment of upper eyelid TT in Amhara Region, Ethiopia, which has been reported separately [12]. In brief, individuals aged 18 years or more with previously un-operated major trachomatous trichiasis (>5 trichiatic lashes) of the upper eyelid were recruited through surgical treatment campaigns in rural areas. Trichiasis was considered to be due to trachoma in the absence of another obvious cause for the trichiasis, such as trauma, malignancy, involutional changes or severe blepharitis.

Following the baseline pre-operative clinical assessment (described below), participants were randomised to one of two surgical intervention groups, which differed only in the type of sutures that were used. The posterior lamellar tarsal rotation procedure was used in all cases to correct upper eyelid TT, using the randomly allocated suture material: (1) PLTR with 4/0 silk sutures (Mersilk, Ethicon) or (2) PLTR with 5/0 polyglactan-910 (Vicryl undyed, Ethicon) [8], [12]. The PLTR was chosen because this is the standard procedure used in Amhara Region. Five general nurses performed all the surgery. They had been trained in TT surgery by the Amhara Regional Trachoma control programme, and had been performing surgery regularly for more than 1 year. They were selected on the basis of their good performance and underwent additional refresher and standardisation training, under the supervision of a senior Ethiopian ophthalmologist [12]. Postoperatively, the operated eye was padded for a day and then tetracycline eye ointment was self-administered twice a day for two weeks.

Participants were re-examined at 6, 12, 18 and 24 months after surgery. To be eligible for analysis in this study a complete follow-up series was required; cases and controls were only selected from those individuals who were examined on all occasions. Individuals who developed any recurrent TT (defined as one or more lashes touching the eye) at any point during the course of the two-year follow-up period were designated recurrent TT “cases” and those without any trichiasis recurrence or evidence of epilation at any time point were designated non-recurrent “controls”. We further limited recurrent TT cases to those that had 3 or more lashes touching the eye at some point during follow-up, as this provided just over 100 recurrent TT cases for analysis. The non-recurrent controls were selected at random from operated individuals who had not developed recurrent TT at any time point during follow-up; these controls were frequency matched with the recurrent cases for age and baseline trichiasis disease severity (determined by the number of lashes touching the eye before surgery).

### Clinical assessment and sample collection

At all assessments participants were examined for clinical signs of trachoma, graded using 2.5× binocular loupes according to the detailed WHO trachoma grading system [23]. The baseline (pre-surgery), 12-month and 24-month follow-up examinations were conducted by a single ophthalmologist (SR); the 3, 6 and 18-month examinations were conducted by a single ophthalmic nurse (EH). Recurrent trichiasis was defined as one or more lashes touching the eye. In preparatory exercises the examiners were standardised to each other and showed strong agreement for the presence of trichiasis (kappa = 0.86). The number of lashes touching the eye in primary position was counted. Individuals with clinical evidence of epilation were considered to have recurrent trichiasis, even if no lashes were touching the globe on examination. Significant conjunctival inflammation was defined as the presence of papillary inflammation grades P2 or P3 of the detailed WHO Trachoma Grading System [23].

Conjunctival swab samples were collected by the examiner from the upper tarsal conjunctival surface for RNA isolation at all time-points, except 3-months. The ocular surface was anaesthetised with preservative-free proxymetacaine 0.5% eye drops (Minims, Chauvin Pharmaceuticals). A dacron polyester-tipped swab (Hardwood Products Company) was passed horizontally across the conjunctival surface 4 times with a quarter turn between each pass. Swabs were then placed directly into a tube containing 0.3 ml of RNA*later* (Life Technologies). Samples were kept on ice packs until frozen later the same day at −20°C.

### Quantitative RT-PCR

The abundance of nine selected transcripts was estimated by quantitative RT-PCR. The choice of targets was informed by our previously published conjunctival transcriptome analysis conducted on pre-operative samples from TT patients in the same population and matched normal controls [5]. These targets broadly fall into three groups: (i) pro-inflammatory cytokines, chemokines or effector molecules (interleukin-1β [*IL1B*], tumour necrosis factor-α [*TNFA*], psoriasin-1 [*S100A7*], chemokine (CXC) ligand-5 [*CXCL5*], nitric oxide synthase-2A [*NOS2A*]), (ii) various matrix metalloproteinases (*MMP7, MMP9* and *MMP12*) and (iii) connective tissue growth factor (*CTGF*).

Total RNA was extracted from the swab samples using the RNeasy Micro Kit (Qiagen). Reverse transcription was performed using the QuantiTect Reverse Transcription Kit (Qiagen) according to the manufacturer's instructions. Multiplex real-time quantitative PCR was performed on a Rotor-Gene 6000 (Corbett Research, Cambridge, UK) using the QuantiTect Multiplex NoROX Kit (Qiagen), according to the manufacturer's instructions. Multiplex assays of up to four separate targets (including *HPRT-1* as the reference gene) were designed by Sigma Life Science (www.Sigma.com/designmyprobe) using Beacon Designer 7.60 (Premier Biosoft International, Palo Alto, CA, USA). The thermal cycle protocol used the following conditions: 95°C for 15 minutes, followed by 45 cycles of (1) denaturation at 94°C for 30 seconds, (2) annealing and extension at 60°C for 30 seconds. Fluorescence data was acquired at the end of each cycle. The relative efficiency of the component reactions was assessed using standards containing all targets in a sequence of tenfold serial dilutions. Reactions were performed in duplicate, in a total volume of 25 µl, which contained 2 µl of sample or standard. Probe and primer sequences are available on request.

### Analysis

We aimed to analyse sample sets from at least 100 cases and 100 controls. This sample size was estimated to have 80% power and 95% confidence to detect a factor with an odds ratio of 2.5 that is present in 50% of the controls. The maximum number of lashes touching the eye during follow-up was used to stratify the recurrent TT group.

The transcript abundances for genes of interest were standardised relative to that of *HPRT-1* in the same reaction using the ΔΔC_T_ method and were successfully normalised by log_10_ transformation [24]. This corrects for variations in the total RNA collected by the swabs. The only exception was *CTGF*, which was not possible to multiplex with these other targets; therefore the quantitation for this target relative to *HPRT* is based upon standard curve analysis.

Data were managed in Access (Microsoft) and analysed in STATA 11 (StataCorp). Cases and controls were comparable in terms of age, sex and pre-operative trichiasis severity, therefore, the relative level of expression of each target was compared between them using unadjusted unpaired *t* tests. Multivariable linear regression models were fitted for the expression level of each target at baseline and the following potential explanatory variables: sex (female), age (in years), case-control status, conjunctiva inflamed (P2/P3) at baseline and the number of lashes touching the eye at baseline. A stepwise selection process was performed to fit each model, retaining terms if the *p*-value for omission was <0.2 and their *p*-value in the model was <0.2, with *p*-values assessed by the likelihood ratio test. Similar models were fitted for each follow-up time point. There was no evidence of a differential effect over time, assessed by likelihood ratio testing with and without an interaction with time (p-values 0.19 to 0.74). Therefore, we combined the data from the four follow-up time points, using random-effects linear models. An un-conditional logistic regression model was developed for the association between recurrent trichiasis and baseline conjunctival inflammation and the expression levels of all nine genes. Again a stepwise selection process was performed to fit each model, retaining terms if the *p*-value for omission was <0.2 and their *p*-value in the model was <0.2, with *p*-values assessed by the likelihood ratio test. The correlation in the expression of the various targets at each time point was investigated using biplots and partial correlation coefficients. To adjust for multiple comparisons, we calculated critical significance thresholds for each table using the conservative Bonferroni correction. Although we make several comparisons, these are un-likely to be truly independent of each other, as one would expect some of these genes to interact in biological networks.

## Results

### Participant selection and characteristics

A total of 1300 individuals with major TT were recruited into the trial and received trichiasis surgery [12]. As there was no difference in the TT recurrence rate between the two alternative sutures, subjects for this nested case-control study were drawn from both trial arms and data combined [12]. In all, 901/1300 (69.3%) were assessed at all five time-points. Only those seen and sampled on all five occasions were eligible for this nested study. Comparing the eligible and excluded people: there was no difference in the proportion who were female (p = 0.3), the eligible group was slightly younger (49 years vs. 51 years, t-test p = 0.02) and had slightly more severe baseline trichiasis (mean number of lashes: 8.0 vs. 7.3, Wilcoxon rank-rum p = 0.006 There were 122 people (seen and sampled five times) who developed recurrent trichiasis of the upper eyelid with three or more lashes touching the eye during the follow-up period; these participants were designated as recurrent “cases”.

There were 740 people (seen and sampled five times) who had no evidence of recurrent TT at any point during the follow-up period. Overall, the non-recurrent group tended to be younger (mean age 48.3 years, compared to 54.1 years, t-test p<0.0001) and had less severe pre-operative trichiasis (mean number of lashes 7.1, compared to 13.8 lashes, Wilcoxon rank-rum p<0.0001) compared to the recurrent TT cases. To ensure that the non-recurrent “control” group was of a similar age and preoperative trichiasis severity, 122 individuals were randomly selected from the potential 740, constrained by frequency matching from within the same combination of age group (18–29, 30–39, 40–49, 50–59, 60–69, 70+ years) and pre-operative trichiasis severity group (0 (epilating), 1–5, 6–9, 10–19, 20+ lashes) as the 122 recurrent TT cases. The baseline distribution of age and trichiasis severity groups were the same for cases and controls, except in one instance, where there was one too few controls to frequency match (Table 1). There was no difference in gender, age, baseline trichiasis severity or suture randomisation arm between cases and controls (Table 2). All cases and controls had tarsal conjunctival scarring at baseline. However, individuals who subsequently developed recurrent trichiasis were more likely to have had tarsal conjunctival inflammation (P2/P3) than the controls pre-operatively.

**Table 1:** Baseline distribution of age groups and trichiasis severity groups for both cases (Ca) and controls (Co).

Age Group (Years)	Total number of lashes											
	0 (epilating)		1–5		6–9		10–19		20+		Total	
	Ca	Co	Ca	Co	Ca	Co	Ca	Co	Ca	Co	Ca	Co
18–29	0	0	0	0	0	0	2	2	2	2	4	4
30–39	0	0	5	5	5	5	3	3	2	2	15	15
40–49	3	3	9	9	5	5	10	10	4	4	31	31
50–59	2	2	6	6	15	15	3	3	10	11	36	37
60–69	2	2	11	11	4	4	6	6	6	5	29	28
70+	0	0	1	1	1	1	3	3	2	2	7	7
Total	7	7	32	32	30	30	27	27	26	26	122	122

**Table 2:** Baseline demographic and clinical characteristics.

	Cases		Controls		P-value
Gender (female)	91/122	(74.6%)	94/122	(77.1%)	0.65
Age (mean)	54.1	(SD 12.6)	53.6	(SD 11.8)	0.75
Lash count (mean)	13.8	(SD 17.0)	14.6	(SD 17.2)	0.79
Suture type (silk)	61/122	(50.0%)	55/122	(45.0%)	0.44
Clinically inflamed (P2/P3)	94/122	(77.1%)	76/122	(62.3%)	0.012

### Samples

A total of 1220 samples had been collected across the five time points from these 244 participants. The nine targets were measured in each of these. There were a small number of samples from each time point for which it was not possible to perform quantitative PCR due to an inadequate RNA sample (Baseline four samples; 6-months three samples; 12-months eleven samples; 18-months six samples; 24-months four samples). For the purpose of this analysis these samples are treated as missing data.

### Gene expression analysis

There was no significant difference between the two trial arms in the expression of any of the targets at any of the time-points. Therefore, the data are not adjusted for trial arm. Recurrent TT was consistently associated with about a 2-fold increase in *S100A7* (at the 0.05 level), on 4 out of 5 occasions. No other targets had a consistent association with recurrent TT (Table 3). Clinically apparent conjunctival inflammation (P2/P3), irrespective of whether there was recurrent TT or not, was consistently associated with increased expression of pro-inflammatory factors (*S100A7, IL1B, CXCL5*) and matrix metalloproteinases (*MMP9, MMP12*) across multiple time points (Table 4). Multivariable linear regression models for each target were fitted for baseline and a combination of the follow-ups (random-effects model), Table 5. Recurrent trichiasis remained significantly associated with increased expression of *S100A7* after adjusting for other factors in these models, both before and after surgery. In an un-conditional logistic regression model for recurrent trichiasis and baseline factors, only conjunctival inflammation and *S100A7* expression were significantly associated with subsequent recurrence (Table 6).

**Table 3:** Gene expression level in recurrent TT cases relative to non-recurrent controls.

Target	Baseline		6-months		12-months		18-months		24-months	
	FC	P-value	FC	P-value	FC	P-value	FC	P-value	FC	P-value
***S100A7***	1.95	0.015	2.05	0.006	1.64	0.08	2.55	0.0012	1.96	0.022
***IL1B***	1.19	0.36	1.02	0.92	1.54	0.014	1.34	0.049	1.07	0.71
***CXCL5***	1.33	0.30	1.04	0.85	1.06	0.76	1.45	0.08	1.04	0.86
***TNFA***	1.05	0.72	1.10	0.42	1.05	0.68	1.17	0.12	1.07	0.62
***NOS2A***	1.21	0.11	1.07	0.59	1.06	0.62	1.18	0.14	1.18	0.30
***CTGF***	1.27	0.22	1.27	0.039	1.12	0.34	1.16	0.22	1.11	0.45
***MMP7***	1.13	0.32	1.18	0.16	1.03	0.80	1.33	0.022	1.16	0.25
***MMP9***	1.17	0.25	1.03	0.82	1.24	0.10	1.38	0.0087	1.23	0.12
***MMP12***	1.02	0.90	1.02	0.90	1.14	0.34	1.42	0.012	1.05	0.74

FC fold change; P values for unpaired *t* test. Using the Bonferroni correction, the critical significance threshold level is a P value of <0.0011.

**Table 4:** Gene expression level in inflamed (P2/P3) individuals relative to non-inflamed individuals.

Target	Baseline		6-months		12-months		18-months		24-months	
	FC	P-value	FC	P-value	FC	P-value	FC	P-value	FC	P-value
***S100A7***	1.87	0.037	2.67	0.013	3.78	<0.0001	2.49	0.0016	4.13	<0.0001
***IL1B***	1.44	0.08	1.85	0.013	2.08	<0.0001	2.03	<0.0001	1.95	0.0009
***CXCL5***	2.27	0.0075	1.75	0.10	2.38	<0.0001	2.16	0.0002	1.66	0.038
***TNFA***	1.03	0.83	1.17	0.37	1.35	0.0088	1.19	0.079	1.09	0.57
***NOS2A***	1.07	0.62	1.44	0.043	1.36	0.013	1.25	0.051	1.29	0.14
***CTGF***	1.31	0.19	1.13	0.49	1.05	0.70	1.10	0.43	1.23	0.16
***MMP7***	1.06	0.67	1.13	0.50	1.11	0.44	1.13	0.32	1.45	0.010
***MMP9***	1.32	0.059	1.35	0.10	1.59	0.0005	1.41	0.0051	1.57	0.0017
***MMP12***	1.46	0.028	1.66	0.0077	1.52	0.0023	1.80	<0.0001	1.51	0.018

FC fold change; P values for unpaired *t* test. Using the Bonferroni correction, the critical significance threshold level is a P value of <0.0011.

**Table 5:** Multivariable linear regression models for the expression of each target at baseline and follow-up.

Target	Baseline		Follow-up	
	FC	P-value	FC	P-value
***S100A7***				
Gender (female)	2.12	0.013	1.89	0.015
Age (years)	-	-	1.03	0.002
Inflammation (P2/P3)	-	-	1.99	<0.001
Recurrent TT case	2.07	0.005	1.78	0.008
Baseline lashes*	1.04	<0.001	-	-
***IL1B***				
Gender (female)	1.49	0.069	-	-
Age (years)	1.01	0.057	1.01	0.001
Inflammation (P2/P3)	1.29	0.194	1.54	<0.001
Recurrent TT case	-	-	-	-
Baseline lashes*	1.03	<0.001	-	-
***CXCL5***				
Gender (female)	1.52	0.205	1.34	0.105
Age (years)	1.02	0.039	1.03	<0.001
Inflammation (P2/P3)	2.05	0.017	1.44	<0.001
Recurrent TT case	-	-	-	-
Baseline lashes*	1.02	0.005	-	-
***TNFA***				
Gender (female)	1.84	<0.001	1.24	0.014
Age (years)	1.01	0.021	1.01	0.001
Inflammation (P2/P3)	-	-	-	-
Recurrent TT case	-	-	-	-
Baseline lashes*	1.01	0.005	-	-
***NOS2A***				
Gender (female)	1.57	0.001	1.40	0.001
Age (years)	-	-	1.02	<0.001
Inflammation (P2/P3)	-	-	1.14	0.036
Recurrent TT case	1.24	0.063	-	-
Baseline lashes*	1.01	0.003	-	-
***CTGF***				
Gender (female)	-	-	-	-
Age (years)	1.01	0.080	0.99	0.008
Inflammation (P2/P3)	-	-	1.18	0.004
Recurrent TT case	1.30	0.156	1.13	0.122
Baseline lashes*	1.02	<0.001		
***MMP7***				
Gender (female)	-	-	-	-
Age (years)	-	-	1.01	0.108
Inflammation (P2/P3)	-	-	1.17	<0.001
Recurrent TT case	-	-	-	-
Baseline lashes*	-	-	-	-
***MMP9***				
Gender (female)	1.44	0.013	1.25	0.027
Age (years)	-	-	1.01	<0.001
Inflammation (P2/P3)	-	-	1.44	<0.001
Recurrent TT case	1.21	0.129	1.14	0.114
Baseline lashes*	1.02	<0.001		
***MMP12***				
Gender (female)	1.58	0.011	1.25	0.031
Age (years)	-	-	1.01	0.001
Inflammation (P2/P3)	1.36	0.066	1.29	<0.001
Recurrent TT case	-	-	-	-
Baseline lashes*	1.02	<0.001	-	-

* Baseline lashes were only included as a potential term in the baseline models.

The four follow-up assessments were combined using random-effects models. Terms were only retained in the models if their p-value was <0.2, by likelihood ratio testing.

**Table 6:** Unconditional logistic regression model for baseline factors that associated with recurrent trichiasis.

Variable	OR	95% C.I.	p-value
Inflammation (P2/P3)	1.81	1.02–3.21	0.041
*S100A7* expression	1.36	1.03–1.81	0.033

Only baseline inflammation and *S100A7* expression level met the criteria to be retained.

**Figure 1:**
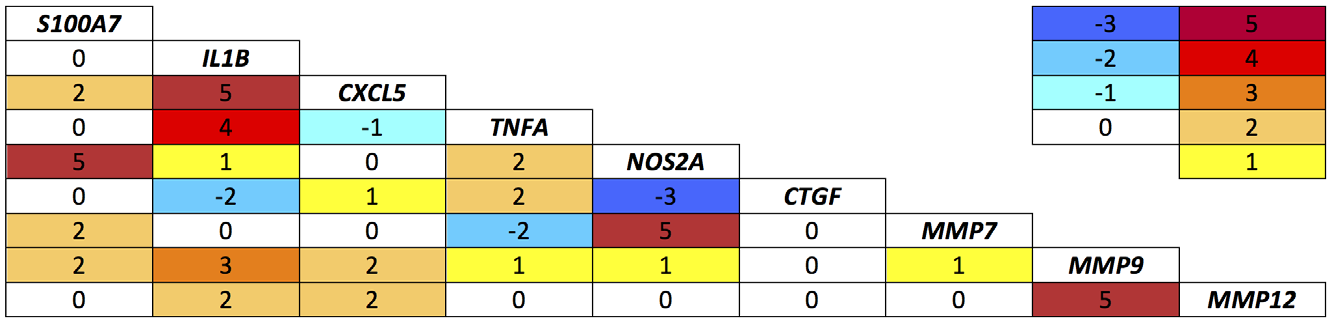
Partial correlation matrix of gene expression. Number of occasions (maximum 5) on which the partial correlation coefficient of expression of each target relative to the other were positively or negatively correlated at the p<0.05 level.

Partial correlation coefficients were calculated for each target with each other for each time point. The total number of significant partial correlations at the 0.05 level is represented in Figure 1. Consistent associations were found on 4 or 5 occasions between (1) *S100A7* and *NOS2A*, (2) *IL1B* and *CXCL5*, (3) *IL1B* and *TNFA*, (4) *NOS2A* and *MMP7*, and (5) *MMP9* and *MMP12*.

## Discussion

Recurrent trichiasis is a challenging problem for prevention of blindness programmes as it renders the surgical intervention less effective, increasing the risk of sight loss. Multiple factors likely contribute to recurrence, which can broadly be subdivided into disease related and surgery related factors. Refinements of the operative procedure and improvements in surgical quality are anticipated to lead to a reduction in early recurrent trichiasis [2], [14]. However, the risk of recurrence is also related to the severity of pre-operative disease and probably individual variations in early wound-healing events and therefore attributable to the underlying cicatricial disease process [2], [12], [25]. In this study we aimed to identify immuno-fibrogenic factors associated with recurrent TT developing over a two-year period.

The choice of targets for this longitudinal gene expression study was guided by findings from the baseline case-control study of the conjunctival transcriptome, which compared individuals with established TT to people without disease from the same population [5]. This earlier study used a two-stage process to identify potential disease biomarkers and pathways: (1) transcriptome-wide microarray analysis, followed by (2) quantitative PCR in a large sample set to confirm findings. This identified a variety of pro-inflammatory (*IL1B, TNFA, S100A7*, *CXCL5, NOS2A*) and tissue remodelling factors (*MMP7, MMP9* and *MMP12*), which were associated with conjunctival scarring and trichiasis before surgery. Therefore, these were chosen as candidates to be investigated in this longitudinal study of recurrent trichiasis following surgery.

In the present study the only gene found to have consistently increased conjunctival expression (both before and after surgery) in individuals who developed recurrent trichiasis was *S100A7* (Psoriasin). There was an approximately 2-fold increase in its expression on four out of the five time-points; this association remained significant after adjustment was made for other potential explanatory factors. Clinically visible conjunctival inflammation (P2/P3) was also associated with increased expression of *S100A7*. Multivariable models indicated that the associations between recurrent trichiasis, *S100A7* expression and inflammation were independent. Unsurprisingly, the expression of several pairs of factors showed a degree of association on multiple occasions.

We previously found *S100A7* to be the most strongly up-regulated gene in the conjunctiva in TT cases relative to normal controls, in the same Ethiopian population and in Tanzanians with conjunctival scarring in the absence of TT [5], [26]. The increase was more marked when the conjunctiva appeared inflamed (P2/P3) [5], [26]. We have also found *S100A7* expression to be significantly increased in children with signs of clinically active trachoma [21]. However, it is difficult to determine in this longitudinal study following trichiasis surgery the potential contribution that S100A7 might make to the pathophysiology of recurrent trichiasis or progressive conjunctival scarring in general. At present there are no suitable animal models for this scarring process that can be manipulated to investigate its potential role.

Psoriasin is a member of the S100 family, a diverse group of calcium binding, low molecular weight proteins, which are expressed in multiple tissues, particularly epithelial surfaces [27]. S100A7 was initially identified in psoriatic skin lesions, and linked to the inflammatory pathophysiology of the disease [28]. In healthy skin it is expressed only at minimal levels. Subsequently, it was found to be increased in other inflammatory skin conditions [27]. A number of epithelium-associated malignancies have increased expression of *S100A7*: carcinoma of the breast, lung, prostate, stomach and cutaneous squamous cell carcinoma. Aggressive tumour behaviour (invasion and metastasis) has been particularly associated with its expression [29]–[33]. S100A7 appears to mediates these effects through increasing inflammation and activation of MMPs, which have parallels in trachoma [34], [35]. More recently, S100A7 has been identified as a potential biomarker for Alzheimer's disease, with marked increases in both cerebrospinal fluid and brain tissue [36].

The functions of S100A7 have only recently begun to be explored in detail. It is an anti-microbial peptide, protecting epithelial surfaces from bacterial infection [37]–[40]. The mechanism by which S100A7 exerts its direct antimicrobial effect is not fully understood; it is possibly through pore-formation, which permeabilises the bacterial cell membrane, or through zinc sequestration [37], [38]. S100A7 also has some chemokine-like actions and may be particularly important in driving an innate immune response. It is chemotactic to both neutrophils and T lymphocytes [41]–[43]. It has been shown to stimulate neutrophils to produce various pro-inflammatory factors (TNFα, IL-6, IL-8, CCL3, CCL4, CCL20), produce reactive oxygen species (ROS) through NADPH oxidase, and to degranulate, releasing myeloperoxidase [44]. S100A7 has also been shown to cause keratinocytes to produce cytokines that probably promote Th1 and Th17 responses (TNFα, IL-1α, IL-23, MIP2, RAGE) [43]. Interestingly, stimulation of keratinocytes with a combination of IL-17A, IL-22 and TNFα increased S100A7 production, which may form a feed-back loop, amplifying inflammation in psoriasis and other conditions [43].

Little is known about the regulation of S100A7. Some bacterial components, such as flagellin, trigger increased production of S100A7, probably through TLR5 [45]. In addition, S100A7 production can also be stimulated by pro-inflammatory cytokines (TNFα, IL-1α, IL-6, IL-17, IL-22) [33], [43], [46]. In breast cancer cell lines, IFNγ down-regulates S100A7 expression, through STAT1 transcriptional activity [47]. This may be of relevance to trachoma, as Th1 responses (characterized by increased IFNγ) are thought to be associated with a less scarred outcome, which could conceivably be partly mediated through suppression of S100A7 [48].

Chronic conjunctival inflammation is an important component of the pathophysiology of trachoma and it is a relatively frequent finding in people with cicatricial disease [2], [49]. Repeated or persistent inflammation in childhood and early adult life is associated with scarring complications later in life [50], [51]. The expression of *IL1B, CXCL5, MMP9* and *MMP12* were increased in the presence of clinical inflammation on several occasions. We have previously reported associations between *IL1B* and *MMP9* and conjunctival inflammation following surgery [20]. These factors are plausibly involved in inducing and regulating inflammation in the conjunctiva and may contribute to the underlying disease process, although in this study they were not associated with recurrent trichiasis. The expression of *TNFA* was not consistently associated with inflammation (only one occasion), which is a pattern we have found in two earlier studies [5], [20].

This study has a number of potential limitations. The outcome of interest was recurrent trichiasis. This can be a variable sign especially if the patient is practicing epilation. However, careful note was made of any evidence of epilation on examination. Secondly, measuring the expression of a gene does not necessarily equate to functional activity of its protein product. Positive findings will need further validation with alternative approaches, such as immunohistochemistry to detect the protein. We did not perform PCR testing for *C. trachomatis* because in this population (which has an active azithromycin mass distribution programme) at baseline we have previously reported that infection was very rare in adults with and without TT (0.1%) and therefore considered it unlikely to be informative [5].

Anti-scarring therapies are increasingly used in ophthalmic surgery to limit cicatricial complication [52]. For example, specific inhibitors of TGFβ and MMPs are being investigated for use in glaucoma filtration surgery. We did not identify obvious potential therapeutic targets, such as a MMPs associated with recurrent trichiasis. It is possible that the MMPs play a role in the outcome of early post-operative wound healing events, although this was not the focus on this study. The observation that increased expression of *S100A7* was consistently associated with recurrent trichiasis indicates that it may have a role in this disease process. In the light of new information becoming available about the contribution of S100A7 to other diseases, our findings suggest that this molecule warrants further investigation in trachoma.
