# Correction: Post-Operative Recurrent Trachomatous Trichiasis Is Associated with Increased Conjunctival Expression of *S100A7* (Psoriasin)

**DOI:** 10.1371/annotation/e55f440e-5c3a-4a69-810c-cd41b69797bf

**Published:** 2013-09-17

**Authors:** Matthew J. Burton, Saul N. Rajak, Athumani Ramadhani, Helen A. Weiss, Esmael Habtamu, Bayeh Abera, Paul M. Emerson, Peng T. Khaw, David C. W. Mabey, Martin J. Holland, Robin L. Bailey

The sixth author's name is misspelled. It should read Bayeh Abera

